# Intravesical Agents in the Treatment of Bladder Clots in Children

**DOI:** 10.3390/pediatric15020024

**Published:** 2023-04-14

**Authors:** Marcello Della Corte, Erica Clemente, Elisa Cerchia, Sabrina De Cillis, Enrico Checcucci, Daniele Amparore, Cristian Fiori, Francesco Porpiglia, Simona Gerocarni Nappo

**Affiliations:** 1 Division of Urology, Department of Oncology, School of Medicine, San Luigi Gonzaga Hospital, University of Turin, Regione Gonzole 10, 10043 Orbassano, Italy; 2 Division of Pediatric Urology, Regina Margherita Hospital, 10126 Turin, Italy; 3 Department of Medical Sciences, University of Turin, Via Verdi 8, 10124 Turin, Italy; clemente.erica@outlook.com

**Keywords:** bladder clots, children, pediatric urology, intravesical agents, blood clots, bladder instillation, endocavitary

## Abstract

Bladder blood clots represent an infrequent urinary condition in children. They usually result from hematuria with many underlying causes, such as urinary tract infections and urethral/bladder traumas. Treatment options for clot removal include trans-urethral or suprapubic bladder irrigation and, if unsuccessful, endoscopic management under general anesthesia with a resectoscope. In younger male children, however, the repeated passage of a trans-urethral resectoscope may be challenging and traumatic, due to the small lumen diameter. Eventually, an open surgical approach can be required in many patients. Few anecdotal non-surgical approaches have been proposed for the management of bladder blood clots in children. This review aims to summarize the conservative techniques described in the literature with the instillation of intravesical agents, analyzing the different strategies and their advantages.

## 1. Introduction

Bladder clot retention depicts an infrequent condition in children resulting from massive and severe hematuria. Once glomerulopathies have been excluded, children and adolescents usually develop hematuria due to benign conditions: urinary tract infections (UTIs), benign urethrorrhagia, trauma, and stones [1]. In infants, causes of hematuria are viral and bacterial UTIs, renal vein thrombosis, renal corticomedullary necrosis, autosomal recessive polycystic kidney disease, adrenal hemorrhage, renal and bladder neoplasms (Wilms tumor or hemangiomas), obstructive uropathy and ECMO support [2]. In children with bone marrow transplantation, hemorrhagic cystitis is presently an infrequent complication, however, it can occur with dramatic violence and may be life-threatening [3].

Bladder blood clots can be diagnosed either during hematuria or once the urine has cleared. They are usually associated with the suprapubic sense of weight, bladder or abdominal discomfort, and dysuria, while in more severe cases they can cause urinary retention, increased creatinine, cystospasm, and worsen hemorrhage [4].

Currently, no official guidelines have been published regarding the management of bladder blood clots, neither for pediatric nor for adult patients.

The diagnostic work-up should begin with an accurate assessment of the clot burden. Ultrasonography is the first mandatory diagnostic imaging, while low-dose computed tomography (CT) should be preferred in case of trauma [5,6].

Manual bladder irrigation through a trans-urethral catheter allows progressive urine clearing and clot aspiration. Large-bore catheters are preferred in order to achieve clot passage [7]. Several alternatives have been proposed in adult patients: Aydin et al. placed a thoracic catheter instead of a urethral one, due to its larger caliber, and they cut the tip side to create a larger suction hole [8]; Jiang et al. preferred a metal urinary catheter [9]; Plawker and Hasmat used a rectal tube [10]. Mesfin et al. tested a 22 Ch prototype catheter designed with more holes than a conventional one, to cover a larger suction area [11]. All these studies lack a control group, therefore their results are not comparable. While in adults a protocol for manual bladder irrigation has been carried out [12], in pediatric patients a standardized approach has never been provided.

If manual irrigation fails, an endoscopic approach, surgical intervention, or intravesical therapy represent possible alternatives for the management of blood clots [2]. The preferable choice depends on the patient’s characteristics (age, clinical conditions, anatomical anomalies), hospital equipment availability, and surgeon’s experience.

Among endoscopic procedures, authors have described direct Ellik suction [13] or variations of the technique, such as the use of a resectoscope or morcellation device to achieve a progressive reduction of clots and facilitate Ellik suction [4,14,15]. Nevertheless, the bladder distension that occurs during the procedure represents a risk factor for bladder rupture [13]. Other authors, in case of Ellik failure, have proposed continuous mechanical suction [16,17,18]. In women, in rare cases, small blood clots may be spontaneously expelled thanks to an increased fluid intake, due to a shorter and wider urethra in females [19]. When urethral catheterization is impossible (e.g., trauma, children), a percutaneous suprapubic tube or endoscopy may be considered [20] and may improve and speed up the conventional trans-urethral technique [21].

In children, the small diameter of the urethra limits the utilization and efficacy of trans-urethral instruments, either catheters or endoscopes [22]. Therefore, in case of failure of the above-mentioned conservative approaches, the atraumatic intravesical instillation of substances may represent an intriguing strategy to achieve clot dissolution, before considering open surgical approaches under general anesthesia.

This review aims to evaluate the use of intravesical agents in children for the lysis of bladder clots.

## 2. Materials and Methods

We searched PubMed, Scopus, Embase, and Web of Science for the management of bladder blood clots through intravesical agents in children. The search terms used were “bladder clot”, “vesical clot”, “child”, “children”, combined with the Boolean operator “AND”. No additional filters were used: all the scientific literature produced in English and Italian up to 15 March 2023 was considered. All work types were evaluated. Two reviewers (M.D.C. and E.C.) selected the eligible studies.

Once identified, we selected the papers using the following inclusion criteria: works based on pediatric patients (age < 18 years), diagnosis of bladder blood clots, and at least one management strategy based on intravesical agents administration.

## 3. Results

One case series and two case reports were obtained, resulting in a total of four children treated with intravesical agents administration (Table 1). Due to the rareness of the patients with bladder blood clots managed in this manner, the research found regarding this topic was limited. Given the scarce results, in order to pursue a more comprehensive overview, we decided to include also 10 studies published on the same topic and not belonging to the pediatric setting (Table 2). For one of them [23], only the abstract was available and the recoverable data were considered. Among the non-pediatric papers, we also included three case reports about animals: two concerning two dogs [24,25] and one about a cat [26].

In the pediatric setting, the first description of the management of bladder clots by intravesical drug instillation is a case series by LaFave et al. published in 1993 [27]. They reported two patients successfully managed with intravesical urokinase. The first one, a five-year-old boy, underwent trans-catheter instillation of 500,000 units of urokinase in 20 cc of saline with a 2 h indwelling time. The procedure was then repeated with 750,000 units in 30 cc of saline. The second patient, aged three weeks, underwent an instillation of 5000 U in 1 cc of saline, repeated at 6 h intervals for four equal doses. Three years after, Korkmaz et al. described their successful experience with streptokinase in a 12-year-old patient who had developed hematuria and bladder clots following a renal needle biopsy. Here, 30 mL of a solution composed of 100,000 units of urokinase plus 100 mL of saline were injected trans-catheter and left in the bladder for 30 min. Repeating the procedure three times every 4 h resulted in clot evacuation [28]. The most recent experience reported by Olarte et al. in 2001 depicts a patient aged three days, treated with alteplase [2]. This is a unique case, concerning a newborn undergoing ECMO support: 5 mL of a solution made by 1 mg of alteplase in 10 mL of normal saline was injected through a 10 Ch Foley catheter, which was clamped for 1 h, then a bladder lavage was performed and the procedure was repeated 8 h later.

All these patients did not show adverse effects and blood tests, when executed, were bereft of coagulation abnormalities (Table 3).

Wider experiences have been reported in adults, resulting in 73 patients managed through intravesical agent administration to dissolve blood clots. 

Initially, Hanna presented a case series of 14 patients managed with intravesical streptokinase, with a 92% success rate (13/14) [23], but this was the only clinical experience with this agent. 

Subsequently, hydrogen peroxide was the intravesical substance more frequently used. Firstly, Warlick et al. discussed the potential application of hydrogen peroxide, reporting a case series of two patients [29]. The first was managed in the operatory room: after several failed attempts with the Ellik evacuator, the hydrogen peroxide instillation allowed an easier mobilization of the clot through the Ellik suction. The second case was managed at the patient’s bedside and clot aspiration was achieved with a Toomey syringe. Bagheri et al. also proposed a bedside approach: through a three-way catheter, they infused 100 mL of 0.15% hydrogen peroxide that allowed urine and clot evacuation [30]. Xu et al. presented a retrospective study on a cohort of 27 patients. Here, 3% hydrogen peroxide solution and 0.9% saline solution were mixed 1:5, and 30–50 mL of the resulting composition was instilled through a three-way 20 Ch Foley catheter and indwelled for 3–5 min. All the patients were treated bedside: clots were evacuated in 27/31 patients after 6–10 irrigation cycles, while the remaining four required surgery [18]. Montelongo et al. used a saline solution with 0.3% hydrogen peroxide and an endotracheal 14 Ch suction catheter. The procedures were performed in the operating room with a rigid cystoscope, and 200 mmHg suction pressure was applied to the cystoscope sheath [31].

An anecdotal experience with chymotrypsin was described by Bo et al. [4]. They included in their study 22 male patients, with prompt success in 19 (86%). The procedure consisted of bladder irrigation with 40,000 U of chymotrypsin in 50 mL of 5% sodium bicarbonate. Both injection and suction phases were achieved through a 20–24 Ch Foley catheter. The remaining unresponsive three patients easily and spontaneously urinated the solution and clots. None required surgery or further approaches.

Lastly, a recent work by Nonato et al. in 2023 [32] focused on a tissue plasminogen activator. They treated an 85-year-old patient with 33 mL of a solution composed of 15 U of recombinant tissue plasminogen activator with 90 mL of saline. The indwelling time of 40 min allowed for successful clot evacuation. 

In the veterinary setting, the overall attention has focused only on alteplase application [24,25,26]. Similarly to Olarte et al. [2], Pineda’s group diluted 1 mg of alteplase in 10 mL of saline solution and administered 5 mL of the solution into a dog bladder, for 1 h. The procedure was repeated 8 h later and then six times at one-month intervals, due to frequent hematuria episodes and new clot development. Similar results were reported by Young et al. in a cat [26] through several intravesical infusions of 0.5 mg of alteplase in 10 mL of saline with a 2 h dwell time, repeated at 8 h intervals. Lastly, four doses with the same dosage and 4 h of dwell time at 12 h intervals, enabled Hooi et al. to evacuate bladder clots in a dog [25]. All these approaches in animals were successful and no adverse effects or toxicity were reported.

For a clearer and schematic comprehension of all the adopted strategies see Table 3 and Table 4. Pediatric studies (Table 3) and non-pediatric ones (Table 4) were summarized separately. 

## 4. Discussion

Many authors have discussed the potential role of intravesical agents for bladder clot evacuation, reporting their own clinical experiences. The key rationale is the choice of a substance, injectable through a trans-urethral catheter into the bladder lumen, that exerts destructive activity on clots. Ideal agents reduce clot size and facilitate its evacuation through the catheter or trans-urethra [7]. The chemical or enzymatic activity of the used substances is summarized in Table 5. 

Currently, the injection of these substances into the bladder is not comprised in official guidelines or scientific societies’ recommendations and is therefore off-label. Consequently, their usage is anecdotal, and doses, usage schemes, and modalities are arbitrary. The actual limits to their widespread use are the scarcity of data, the lack of proper validated schemes, and the unknown potential adverse effects. Despite the fact that the papers reviewed in this work did not show significant adverse events, these agents are not devoid of potential side effects.

Looking at thrombolytic agents, they all activate plasminogen to produce plasmin, a pivotal step that degrades factors V-VIII, fibrin, and fibrinogen. The theoretical risk is their potential systemic hemorrhagic effect due to the trans-urothelium absorption. Therefore, if the urothelium is not intact, their application is contraindicated because systemic fibrinolysis may result [27]. In addition, they may worsen bleeding if the hemorrhage is of intravesical origin [25].

Chymotrypsin has demonstrated safety and effectiveness in all the patients treated by Bo et al. It is historically known for its capacity to denature proteins and liquefy clots. The enzyme activity depends on basic environmental pH (optimal value 7.8), therefore alkalinization with sodium bicarbonate is required to achieve enzyme activation [4].

Hydrogen peroxide has been widely used for bladder clot evacuation. Probably, it inhibits adenosine diphosphate-induced platelet aggregation. Some researchers have reported the potential risk of thermal injury on vessels, fibrin thrombi formation, and arteriolar spasm [29], while its free diffusion through the vessel walls may induce the formation of intra-luminal bubbles, embolisms, and vessels obstruction, due to its conversion into water and oxygen [30]; however, such event was not reported in all the presented works.

The first emerging finding of this review regards the very restricted number of pediatric patients treated with this approach, which impedes a direct comparison between the different agents.

Conversely, many centers have tried these procedures in adults, obtaining high and satisfactory success rates. In particular, no adverse events have been reported and the procedure was resultantly safe, efficient, and minimally invasive. Even in the veterinary setting, although the described cases are limited, the use of intravesical agents for clot management seems to be promising.

Furthermore, the use of intravesical agents does not prejudge any subsequent endoscopic or surgical strategies in case of failure. Indeed, if a complete clot evacuation is not achieved, they can theoretically facilitate the surgical procedure.

**Figure 1:**
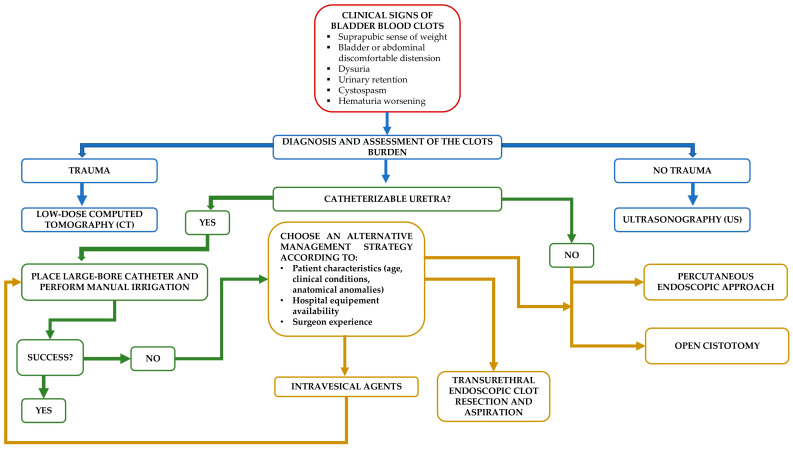
Flow chart of clinical assessment and management of bladder clots.

In light of the above, we wish to summarize our strategy to manage bladder blood clots (Figure 1).

A patient with recent or current hematuria presenting with suprapubic discomfort, dysuria, urinary retention, bladder overdistension, cystospasm, or worsening of hematuria, should undergo diagnostic imaging. In case of previous trauma, a timely low-dose computed tomography (CT) is recommended, while abdominal ultrasonography (US) is chosen in all the other cases.

Once the diagnosis of bladder blood clots is carried out, the first approach is to place the wider possible trans-urethral catheter and perform manual irrigation. This procedure is successful in most cases. In case of failure, an alternative management strategy must be chosen, according to the patient’s clinical characteristics, the medical equipment availability, and the surgeon’s experience, choosing from intravesical agents to endoscopic or surgical approaches. In our opinion, the adoption of intravesical agent instillation together with manual irrigation represents a feasible option in all settings (bedside or operating room), which is minimally invasive and avoids the need for general anesthesia. Only if this approach fails, a more aggressive strategy with an endoscopic, percutaneous, or surgical approach can be pursued. 

In the rare cases of the not-catheterizable urethra (e.g., trauma, previous bladder neck surgical closure, augmented bladders [34]), the percutaneous approach and open cystotomy represent the eligible strategies.

### 4.1. Why Consider Intravesical Agents Strategy?

Among the theoretical advantages, we undoubtedly specify general anesthesia avoidance, since the proposed strategy may be executed on awake patients due to its low invasiveness. Pain relief should always be considered, preferring virtual reality analgesia [35], nitrous oxide [36], or other sedation techniques [37]. 

The relative safety of exclusive intravesical usage allows one to empirically exclude any systemic adverse effects, relying on a circumscribed bladder activity, although the scarce experience and the limited number of treated patients would disguise hypothetical infrequent adverse effects. 

The easiness of the procedure allows for prompt and quick treatment in all contexts, considering both the patient (e.g., not transportable patients, ECMO, dialysis) and the healthcare side (e.g., ambulatory, emergency department, peripheral hospital, absence of pediatric specialists). 

The relative cheapness of the majority of the substances and equipment required reduces surgical management-related costs. 

The success rate reported so far is elevated and promising. 

For the reasons mentioned above, the procedure is promptly repeatable if unsuccessful or in case of relapse and upfront surgery remains a valid remedy. 

**Scheme 1:**
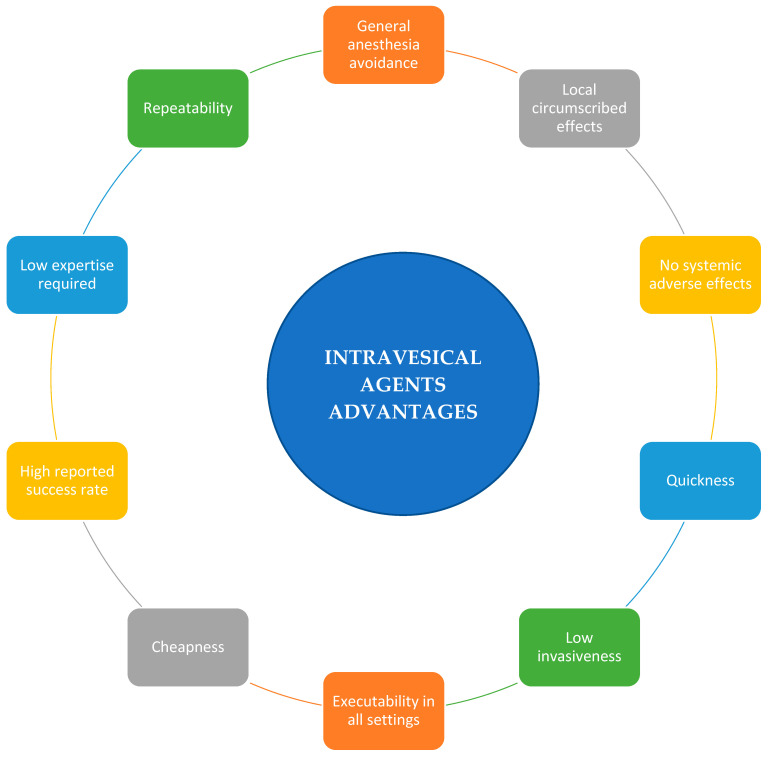
Synthesis of all the advantages of intravesical agents use for bladder clot evacuation.

Lastly, considering the relative easiness of the procedure, a high expertise level is not required (Scheme 1).

However, it has to be borne in mind that the use of such agents is off-label and data in the literature are scanty and of poor quality.

### 4.2. Limits of This Review

We strongly encourage all physicians to achieve a full evaluation of both patient and environmental characteristics before proceeding with an intravesical agent administration. This review analyzed the restricted number of works published on this issue, with an exceedingly limited number of treated patients. The works focused on pediatric patients consisted only of case series and case reports, that conceptually play a key role mainly in stimulating hypotheses and new developments [38]. The retrospective and prospective analyzed studies did not include pediatric patients, therefore a generalization would be improper and arbitrary. We resumed them by offering an objective overview of atypical and off-label medical strategies, whose safety and routine use should be validated through multicenter studies and controlled clinical trials. Actually, the low scientific value of the analyzed works does not allow one agent to be defined as preferable to another one: no clinical trials were found and no case-control studies have been carried out. The overinterpretation or incorrect comprehension of this type of work could lead to “anecdotal fallacy” [38]. The proposed overview of techniques aims to simplify topic comprehension. Nevertheless, for the clinical choice of a specific management strategy, we suggest a comprehensive reading of the corresponding cited work, in particular regarding the urokinase and chymotrypsin single-center experiences. We specify that the intravesical use of the mentioned agents is not included in drug data sheets, therefore it has to be considered an off-label application. 

All the analyzed work covered only native bladders, therefore they did not include experiences with intravesical use on neobladders or augmented bladders [34], whose anatomical characteristics and absorption properties have shown completely different behavior, both in humans and experimental animals [34,39,40,41]. Therefore, the use of these agents in augmented bladders should be avoided. 

## 5. Conclusions

Intravesical agent administration represents a historical but interesting strategy for blood clot evacuation in children with a recent renewed interest. Although their use is limited by anecdotal and scarce experiences, the usages described on adults allow us to reconsider their applicability to children in selected cases. More studies need to be conducted to validate the use of these scarcely invasive and promising therapeutic strategies.

## Figures and Tables

**Table 1:** Summary of the published works concerning pediatric patients. When patients’ single ages were available, they were reported singularly. Legend: Y = years, W = weeks, D = days.

N.	Authors	Year	Type	Number of Patients	Age
1	LaFave et al. [27]	1993	Case series	2	5 Y 3 W
2	Korkmaz et al. [28]	1996	Case report	1	12 Y
3	Olarte et al. [2]	2001	Case report	1	3 D

**Table 2:** Summary of all the remnant published works not concerning pediatric patients. When patients’ single ages were available, they were reported singularly. Legend: NA = not available, Y = years.

N.	Authors	Year	Type	Number of Patients	Age
4	Hanna [23]	1979	NA	14	NA
5	Warlick et al. [29]	2006	Case series	2	47 Y 76 Y
6	Bo et al. [4]	2014	Perspective study	22	Range 42–82 Y
7	Pineda et al. [24]	2015	Case report	1 dog	3 Y
8	Bagheri et al. [30]	2015	Case report	1	49 Y
9	Hooi et Lemetayer [25]	2017	Case report	1 dog	6.5 Y
10	Xu et al. [18]	2020	Retrospective study	31	Range 42–82 (Mean 61.2 Y)
11	Young et al. [26]	2021	Case report	1 cat	5 Y
12	Montelongo-Rodriguez et al. [31]	2022	Case report	2	75 Y 65 Y
13	Nonato et al. [32]	2023	Case report	1	85 Y

**Table 3:** Adopted treatments in papers concerning pediatric patients. The different expressions used to define the same concept (e.g., “prothrombin” instead of “serum coagulation”) reflect the precise data of the reviewed work, where any further clarification would have been arbitrary. Legend: NO = not occurred, Y = years, W = weeks, D = days, S = success, UI = international units.

N.	Author	Patient Age	Agent Used	Dose and Modality	Diagnostic Checks	Adverse Effects	Outcome
1	LaFave et al. [27]	5 Y	Urokinase	500,000 UI/20 mL normal saline. Dwelling 2 h, twice	Stable Prothrombin, partial thromboplastin time, and hematocrit	NO	S
		3 W		5000 UI/1 mL normal saline. Spontaneous micturition. Four doses at 6 h intervals	No bleeding	NO	S
2	Korkmaz et al. [28]	12 Y	Streptokinase	100,000 IU/100 cc of saline, volume infused 30 mL. Dwelling 30 min. Three times at 4 h intervals	Hematocrit, serum coagulation	NO	S
3	Olarte et al. [2]	3 D	Alteplase	1 mg/10 mL normal saline	Not reported	NO	S

**Table 4:** Treatments in published works not concerning pediatric patients. Legend: NA = not available, NO = not occurred, Y = years, S = success.

N.	Author	Age—Animal	Agent Used	Dose and Modality	Diagnostic Checks	Adverse Effects	Outcome
4	Hanna [23]	NA	Streptokinase	NA	NA	NA	13/14 S
5	Warlick et al. [29]	47 Y	Hydrogen peroxide	Intraoperative use: hydrogen peroxide 120 mL 0.15% in normal saline Dwelling 2–3 min.	NA	NO	S
		76 Y		1.5 L of 0.3% hydrogen peroxide in normal saline			
6	Bo et al. [4]	42–82 Y	Chymotrypsin	40,000 UI/50 mL sodium bicarbonate 5%. Dwelling 30 min	NA	NO	19/22 S
7	Pineda et al. [24]	3 Y—Dog	Alteplase	1 mg/10 mL normal saline, volume infused 5 mL. Dwelling 1 h. Twice at 8 h intervals	NA	NO	S
8	Bagheri et al. [30]	49 Y	Hydrogen peroxide	Single shot of 100 mL 0.15% solution	NA	NO	S
8	Hooi and Lemetayer [25]	6.5 Y—Dog	Alteplase	0.5 mg/10 mL normal saline four times. Dwelling 4 h. Repeat at 12 h intervals	Prothrombin and activated partial thromboplastin times remained within normal ranges	NO	S
9	Xu et al. [18]	61.2 ± 10.1	Hydrogen peroxide	3% solution in normal saline (1:5 ratio). Volume infused 30–50 mL. Dwelling 3–5 min	NA	NO	27/31 S
10	Young et al. [26]	5 Y—Cat	Alteplase	0.5 mg/10 mL normal saline. Dwelling 2 h	NA	NO	S
11	Montelongo-Rodriguez et al. [31]	75 Y	Hydrogen peroxide	0.3% in normal saline. Intraoperative irrigation with 1.5 L	NA	NO	S
		65 Y			NA		
12	Nonato et al. [32]	85 Y	Tissue plasminogen activator (tPA)	15 U/90 mL normal saline. Dwelling 40 min	NA	NO	S

**Table 5:** Currently described intravesical agents to dissolve clots.

Agent	Mechanism	Rationale
Urokinase [27]	Enzymatic	Serin-protease plasminogen activator
Streptokinase [23,28]	Chemical	Hydrolytic plasminogen activator
Alteplase [2,24]	Enzymatic	Serin-protease plasminogen activator
Chymotrypsin [4]	Enzymatic	Serin-protease proteolysis Combined with sodium bicarbonate to reach alkaline pH
Hydrogen Peroxide [18,29,31]	Chemical	Protein denaturation through oxidation activity Inhibition of adenosine diphosphate-induced platelet aggregation [29,33]

## Data Availability

Not applicable. No new data were created or analyzed in this study. Data sharing is not applicable to this article.
